# When Love Is in the Air: Understanding Why Dogs Tend to Mate when It Rains

**DOI:** 10.1371/journal.pone.0143501

**Published:** 2015-12-02

**Authors:** Sreejani Sen Majumder, Anindita Bhadra

**Affiliations:** Behaviour and Ecology Lab, Department of Biological Sciences, Indian Institute of Science Education and Research–Kolkata, Kolkata, India; Duke University, UNITED STATES

## Abstract

Seasonality of reproduction is observed in many species of organisms, across taxa, and is influenced by both biotic and abiotic factors. While such seasonality is easy to understand in temperate species exposed to extreme climates, it is more difficult to explain in the tropics. In many tropical species offspring are born during the season of high precipitation, which also coincides with high resource availability. Interestingly, in India, free-ranging dogs seem to mate, and not whelp, when it rains—an observation that cannot be explained by the resource abundance hypothesis. We carried out an extensive study to identify the mating seasons of free-ranging dogs, and observed a strong correlation between both the incidence and frequency of mating related behaviours of dogs, and precipitation levels. There are two clear mating seasons, of which the primary mating season coincides with the monsoon (rainy season) and the secondary mating season coincides with the nor’westerlies in this part of India. We speculate that this strong correlation is an effect of chemistry, rather than biology. While male dogs can mate round the year, females come into estrous seasonally. In the urban environment, dogs are exposed to a lot of olfactory noise, which can dilute the signal present in sex pheromones of the females in heat. A shower leads to increased humidity and reduced temperature of the air, leading to intensification of pheromone signals that trigger a sexual response in the dogs.

## Introduction

Most species of mammals exhibit some degree of reproductive synchrony, so that breeding occurs during optimal conditions [1, 2]. Seasonal breeders successfully mate only during a particular time of the year, typically giving birth to offspring at a time which is ideal for the survival of the young [3]. Seasonality of reproduction is observed in mammals across all latitudes and has even been suggested in the archetypal example of continuously breeding species, *Homo sapiens* [4]. This can be observed in animals, both in terms of the onset of reproductive maturity for young individuals, as well as reproductively active phases for the population in an annual scale [5]. Animals living in seasonally harsh climates tend to display sharply delineated, short breeding seasons, while those that live in relatively non-varying environments display little or no seasonality of reproduction [1]. Seasonality of reproduction can be strongly influenced by abiotic factors like variations of day length, temperature and humidity, which in turn can affect resource availability [6–13]. In fact, it has been shown, at least in the case of primates, that the effects of climate and latitude on birth seasonality are mediated via the availability of resources. Temperate species are more sensitive to changes in day length and temperature, while tropical species are more affected by rainfall patterns. The width of the peak of birth seasonality can be extremely variable, and is often dependent on multiple environmental as well as biological factors [14].

Why do animals in the tropics display seasonality of reproduction? It has been suggested that seasonal breeders are able to maximize fitness in individuals by synchronizing energetically demanding periods of the breeding cycle with periods of maximum food availability or quality, by giving birth just before or during the peak in resource availability [5, 15–21]. Lactation is the most energetically costly phase in the reproductive cycle of female mammals; it is thus beneficial to the female to match this phase with a period of resource abundance, so that she has access to food supply to replenish her lost reserves of energy [1]. This idea is substantiated by observations on many species like giraffes *(Giraffa camelopardalis*), African elephants *(Loxodonta africana*), gazelles, rhesus macaques (*Macaca mulatta*) and wolves (*Canis lupus*) [22–27]. The fact that seasonal breeders can become continuous breeders in resource abundant conditions like domestication and in zoos also lends support to this hypothesis [1, 28, 29]. Though seasonality of reproduction is well understood in temperate species, especially with respect to shift in day length, the mechanism of seasonality in tropical animals is open to exploration. The most accepted hypothesis to explain seasonality of reproduction in the tropics pertains to the maximum availability of resources during the wet season [30–34]. Is the availability of resources the only cause of increased reproductive activities during the wet season in the tropical regions? More importantly, how does rainfall trigger reproductive activity in the tropics?

Dogs (*Canis lupus familiaris*) are the first species to have been domesticated [29], and though most canids are known to be seasonal breeders [27, 29, 35–37] domestic dogs are known to breed continuously, with no clear mating season [38–40]. However, indirect evidence for seasonality of breeding have been suggested [41], especially in free-ranging dogs [42]. In India, free-ranging domestic dogs are a ubiquitous presence in all kinds of human habitats, leading a life of scavengers [43, 44]. It has been observed that the free-ranging dogs in West Bengal, India have a clear mating season, which coincides with the monsoon or the wet season [45, 46]. Monsoon is also the mating season for various other mammals in the Indian subcontinent [30, 47, 48]. This presents a very intriguing paradox—mating, and not whelping, occurs during the monsoon, with the offspring mostly being born during the winter, and so cannot be explained by the hypothesis of resource abundance. The case of the dogs is all the more interesting because being scavengers mostly dependent on wastes and offerings from humans for their sustenance [44, 49], their resources are expected to be constant throughout the year. However, while pet dogs can reproduce aseasonally [50], free-ranging dogs seem to show definite seasonality of reproduction, mating during the monsoon and whelping in the winter. Thus the rains somehow trigger reproductive activities, and this does not apparently provide any adaptive advantage to the breeders.

In this paper we show that not only is there a strong concurrence between precipitation and the mating behaviour of free-ranging dogs, but mating is highly correlated with precipitation levels. We would like to suggest that the proximate cause for this strong correlation lies in abiotic, rather than biotic mechanisms, leading to the seasonal mating habit observed in free-ranging dogs.

## Methods

The Animal Ethics Committee, Indian Institute of Science Education and Research Kolkata provided permission for this work.

The work reported here included two different sampling exercises, one involved a year-long population level census, while the other involved observations of free-ranging dog groups during the monsoon for four years.

### Long term data

We carried out behavioural observations on free-ranging dogs in Kolkata (22.5667° N, 88.3667° E), West Bengal, India from the year 2010 to 2013. The study was conducted from 15^th^ July to 15^th^ October for the first three years, and from 15^th^ June to 15^th^ October for 2013. The period of the study was based on earlier observations of mating during the monsoon and pup occurrence in the winter [46, 51–53]. In West Bengal, monsoon arrives around mid-June, with the thrust beginning in July; monsoon recedes in September—October [54]. A neighbourhood in Saltlake, Kolkata was chosen for the observations, based on convenience and safety of sampling during late hours. Dogs were observed three days a week from 17:00h to 00:00h using instantaneous scans and all occurrences sampling. Each dog group was observed for two hours every day from this above mentioned time period randomly, so that we got 6 hours of data for each dog group per week. We collected data for a total of 954 hours on 67 free-ranging dogs belonging to 12 groups, over 159 days. The area of this study was constant over the four years, but the dogs varied between the years due to natural fluctuations in the population. The precipitation and temperature for each day of observation was recorded from the website of the India Meteorological Department (http://www.imd.gov.in/). At the time of observations, we also made a qualitative note of the weather conditions, like dry, mild drizzle, medium rainfall or heavy rainfall. This qualitative record was later used to quantify precipitation levels into three categories—low (dry—light drizzle), medium (short intense shower and light but steady rain over 2–3 hours), high (heavy and prolonged rain). Table 1 provides an ethogram of behaviours used in this study (also see S1 Fig).

**Table 1:** Ethogram of mating related behaviours (MRB) used in the study.

Behaviour	Description
Genital sniffing (GS)	A male sniffs at the genitalia of the female
Try to clasp (TC)	A male tries to clasp a female from the back
Marking with urine (MK)	Scent marking by leg lifting
Running together (RT)	Running together male and female
Mount (MT)	A male climbs over a female in an attempt to mate; usually leads to copulation

### Year-long census

We selected 40 locations randomly from Kolkata (22.5667° N, 88.3667° E), Kalyani (22.9750° N, 88.4344° E), Kanchrapara (22.9700° N, 88.4300° E), Barrackpore (22.7600° N, 88.3700°E) and Barasat (22.7200° N, 88.4800° E), West Bengal, India covering rural, urban and semi urban areas (S2 Fig). A census of free-ranging dogs was carried out in these locations, covering each of the 40 locations once over a three month period for a year (15^th^ April 2014—14^th^ April 2015). Thus we collected data from 5–10 locations every fortnight, covering each of the 40 locations four times during the year. Each three-month period overlapped, but did not completely coincide with, a season in West Bengal [55]–summer (mid-April—mid-July), monsoon (mid-July—mid-October), winter (mid-October—mid-January), spring (mid-January—mid-April). The time of census was fixed between 0600-0900h and 1600-1900h when dogs are typically seen to be active on streets. We thus had 160 censuses in the whole year from the 40 locations, and an additional set from 5 of the locations within a fortnight (15–30 April 2015) so that the sampling period could be circular for part of the analysis. For the censuses we followed the spot sampling method described in [51]. The observer walked along all roads and by lanes of each location and whenever a dog was sighted, the age category (Adult/Juvenile/Pups) of the dog, it’s group size, sex and the behaviour at the time of sighting were recorded. The weather condition at the time of the census was recorded as described above, and the temperature and precipitation levels for the day were also recorded from the IMD website. We divided the behaviours into two categories, mating-related and non-mating behaviours. Table 1 provides an ethogram of all the mating-related behaviours observed.

### Statistical Analysis

Using the actual levels of precipitation recorded on the day of observations (from IMD), a regression analysis was carried out using data pooled across years to test if the frequency of MRB depended on the precipitation levels. Repeated measures ANOVA were used to test for variation in MRB between years, and between weeks of observation (within a year). We divided the precipitation levels on the days of observation into three categories: high (>20mm per day), medium (10–20 mm per day) and low (0–10 mm per day). Frequency of MRB at different precipitation levels were compared using a Kruskal-Wallis test. A full factorial ANOVA with post hoc Tukey’s test was conducted for the entire dataset, considering the frequency of MRB as the dependent variable, and years, weeks, precipitation levels and behaviours as independent variables. ANOVA with post hoc tests for different levels of precipitation over 12 weeks of 4 years was used to understand the variations at the level of each mating related behaviour. In the year-long census, a repeated measures ANOVA was used to check for variations in dog numbers between seasons in the 40 locations. Kruskal-Wallis tests were carried out to test for the effect of season on the occurrence of MRB. All statistical analysis was carried out in StatistiXL 2007. A distribution fitting exercise was carried out in the software Igor Pro using the frequency of MRB and precipitation data for every fortnight.

## Results

### Long term data

**Fig 1:**
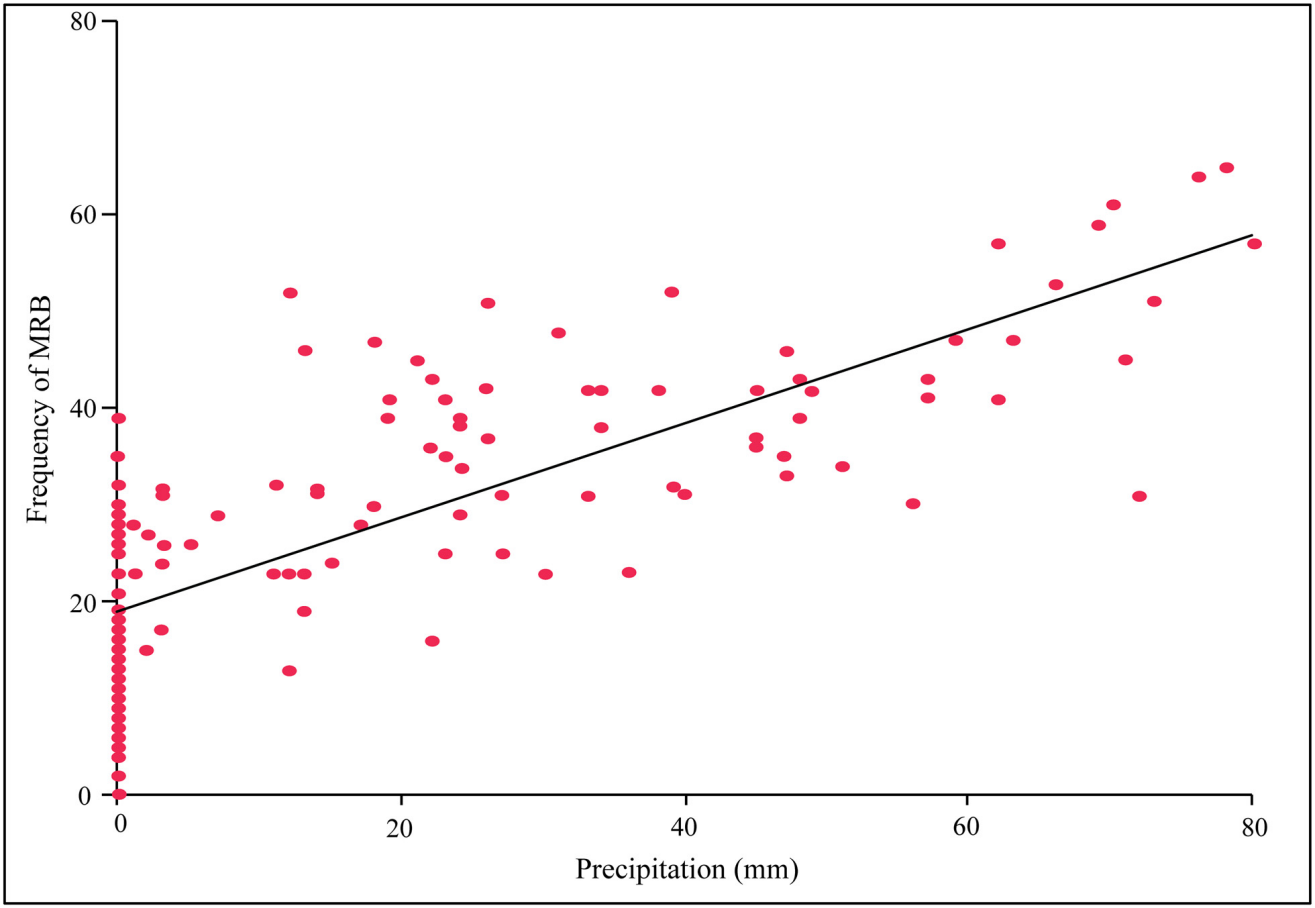
The occurrence of mating related behaviours (MRB) was highly correlated with precipitation levels (mm). The precipitation levels reported here are actual readings of precipitation on the day of observations, as given by IMD. The linear fit is given by the black line, represented by the equation y = 0.4888x + 18.893, R^2^ = 0.6117.

Mating related behaviours (MRB) were observed in all four years during the monsoon [56]. In 2013, MRB were first observed in the month of June, while in all the other years the onset of mating was in the month of July. The occurrence of MRB reduced after the month of September. Hence observations spanned from June to September for 2013, and from July—September for the other three years. The frequency of MRB was strongly dependent on the precipitation levels on a given day (Linear Regression: R^2^ = 0.612, F = 223.694, p < 0.001; Fig 1). There was significant variation in the frequency of MRB across the years when the data for the period July—September was considered (Repeated measures ANOVA, F_3,44_ = 7.696, p < 0.0001). Post hoc analysis revealed that there was significant difference in the frequency of MRB between 2013 and the other three years, while there was no difference in the levels of MRB between 2010, 2011 and 2012 (Table 2). Interestingly, when the frequency of MRB for first three months after the onset of mating were considered, i.e., June to August for 2013 and July—September for the other years, there was no significant difference between the years within the same precipitation category (Repeated measures ANOVA, F_3,44_ = 1.670, p = 0.187). This suggests that the mating behaviour in dogs has some pattern that might be correlated with the precipitation pattern. In both the above cases, there was no significant variation in MRB within a precipitation category when the data was considered at a weekly level (Repeated measures ANOVA, F_11,36_ = 1.151, p = 0.353; F_11,36_ = 1.472, p = 0.185). For all subsequent analysis, the data for the June—August period of 2013. i.e. for the first three months after the onset of mating was considered.

**Table 2:** Results of the post hoc Tukey’s test for Repeated Measures ANOVA carried out to check whether there was significant variation between the years (2010–2013) for the occurrence of mating related behaviours at different levels of precipitation (in mm) for three months from the onset of mating activities (July to September for the first three years, June to August for 2013).

Test	Y Variable	Group 1	Group 2	Mean Diff.	SE	q	Probability
Tukey	>20 mm	2010	2011	6.500	4.446	1.462	0.731
			2012	-2.417	4.446	0.544	0.980
			2013	-4.000	4.446	0.900	0.920
		2011	2012	-8.917	4.446	2.005	0.495
			2013	-10.500	4.446	2.361	0.352
		2012	2013	-1.583	4.446	0.356	0.994
	10–20 mm	2010	2011	4.500	2.132	2.110	0.451
			2012	1.833	2.132	0.860	0.929
			2013	4.667	2.132	2.188	0.419
		2011	2012	-2.667	2.132	1.251	0.813
			2013	0.167	2.132	0.078	1.000
		2012	2013	2.833	2.132	1.329	0.784
	nill	2010	2011	0.333	1.466	0.227	0.998
			2012	-2.833	1.466	1.933	0.527
			2013	-0.583	1.466	0.398	0.992
		2011	2012	-3.167	1.466	2.160	0.430
			2013	0.250	1.466	0.171	0.999
		2012	2013	3.417	1.466	2.331	0.363

**Fig 2:**
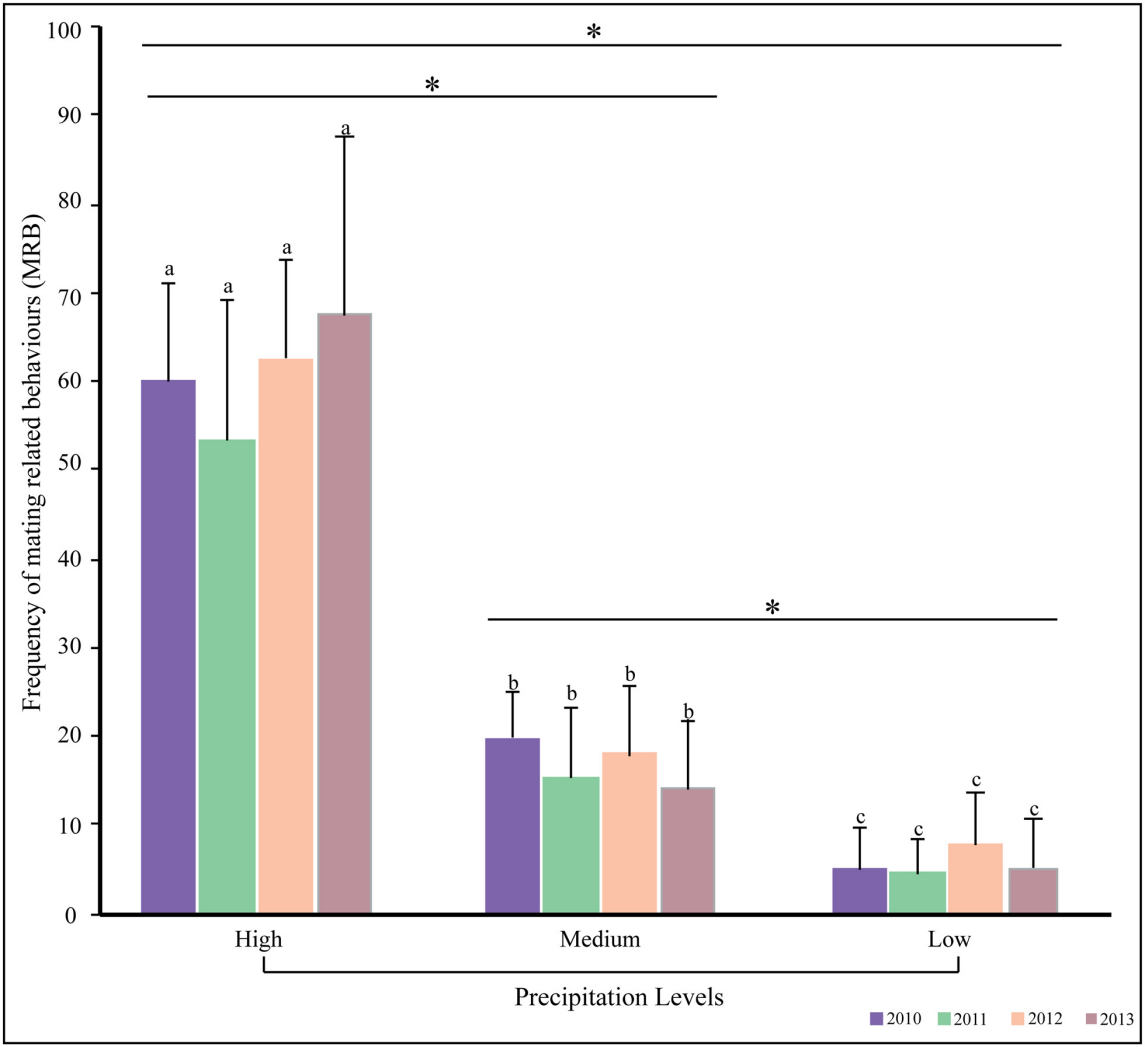
The frequency of MRB varied with precipitation levels. The bar chart shows the mean and standard deviation of the frequency of all MRB occurring at different levels of precipitation (as noted during the time of observations), over four years, 2010 to 2013, during the primary mating season. Variation in the frequency of MRB was not significant across the four years within a precipitation category (alphabets), but varied significantly different levels of precipitation within a year (*).

The frequency of MRB was highest for the “high precipitation” category– 2703 acts of MRB were observed over the four years on days that received high precipitation, while this number was 778 and 252 in the medium and low precipitation categories. The variation in the frequency of MRB for different precipitation categories was significant (Kruskal-Wallis test: χ^2^ = 8.540, df = 2, p = 0.014). The full factorial ANOVA was highly significant (F_59,660_ = 43.082, p < 0.0001), with only the interaction between year and precipitation level being non-significant (Table 3). Post hoc tests revealed significant differences between all behaviours, and between all the three precipitation levels. But variation within the years was not significant (Fig 2, Table 3).

**Table 3:** Results of a Full Factorial ANOVA to test for interaction between precipitation levels, occurrence of MRB and years of observation.

**Overall test of model for Y = Frequency**					
**Source**	**Type III SS**	**Df**	**Mean Sq.**	**F**	**Probability**
Model	27666.682	59	468.927	43.082	**0.000**
Error	7183.750	660	10.884		
Total	34850.432	719			
**Tests of effects for Y = Frequency**					
**Source**	**Type III SS**	**Df**	**Mean Sq.**	**F**	**Probability**
Behaviour	8288.758	4	2072.190	190.380	**0.000**
Precipitation	13874.586	2	6937.293	637.357	**0.000**
Year	92.960	3	30.987	2.847	**0.037**
Behaviour*Precipitation	4117.275	8	514.659	47.284	**0.000**
Behaviour*Year	626.019	12	52.168	4.793	**0.000**
Precipitation*Year	102.769	6	17.128	1.574	0.152
Behaviour*Precipitation*Year	564.314	24	23.513	2.160	**0.001**

**Fig 3:**
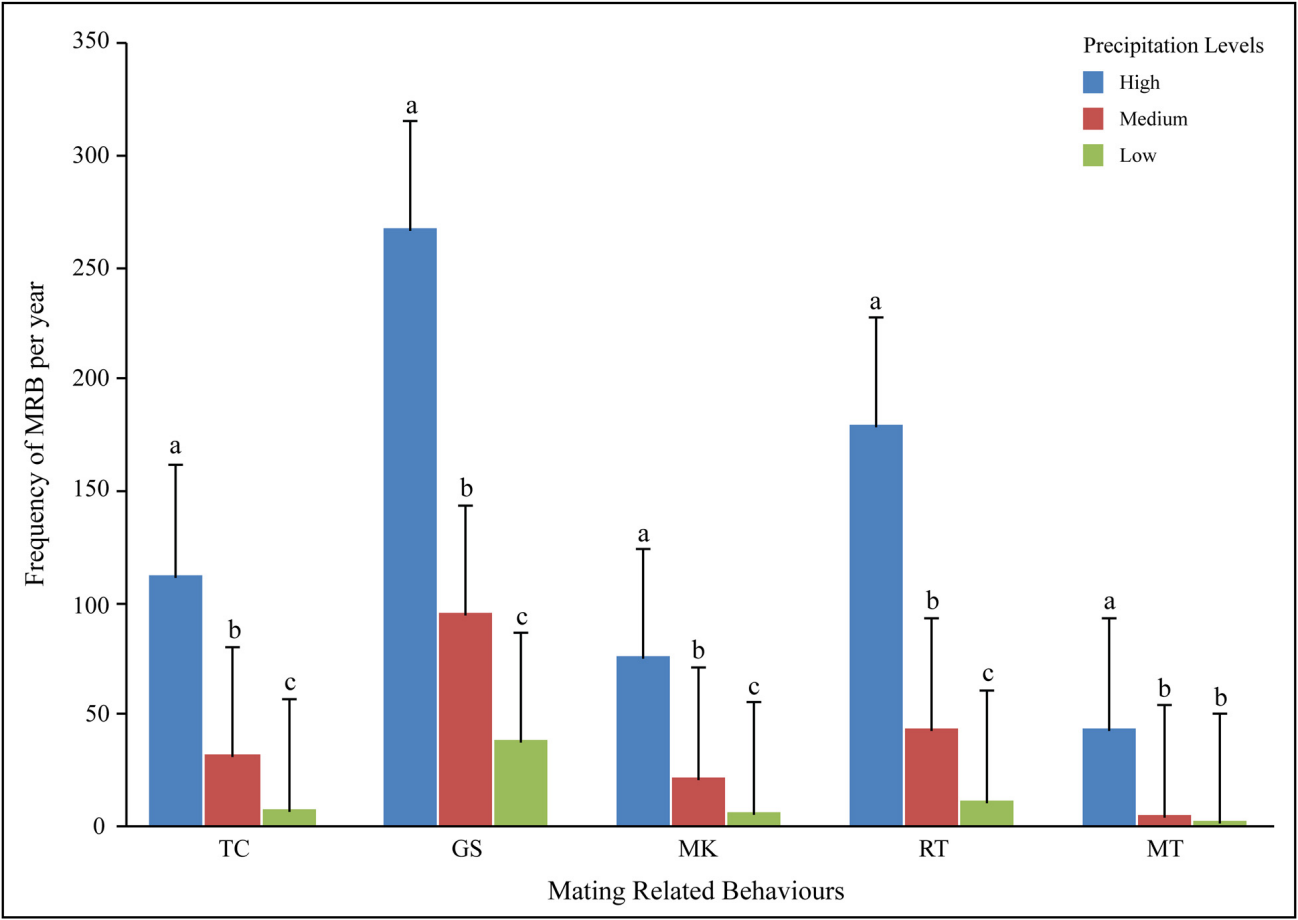
All MRB showed variation across precipitation categories. Mean and standard deviation of the frequency of different mating related behaviours observed at the three precipitation levels noted during the time of observations—the different alphabets represent significant differences within a behaviour category, between precipitation levels.

Since there was significant variation between the behaviours, an ANOVA with post hoc tests at the level of each behaviour was used to test for variations for occurrence of the behaviour at different levels of precipitation. For all behaviours other than MT, the frequency of occurrence of the behaviour was significantly different between all three precipitation levels, while the rate of MT was different only for the high precipitation category (S1 Table; Fig 3).

### Year-long census

**Fig 4:**
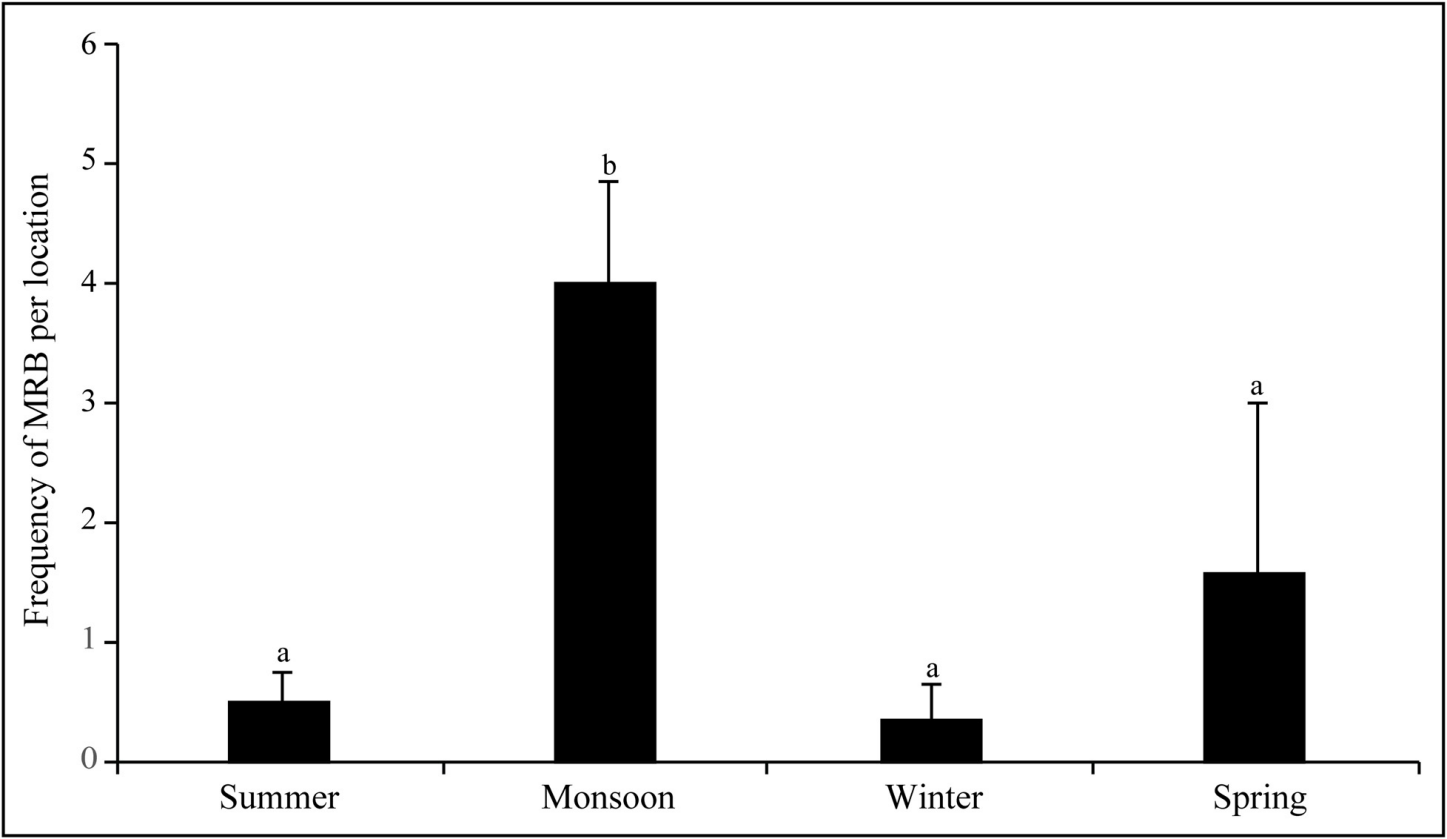
The year-long census showed that highest MRB occur in the monsoon. A bar chart showing the mean and standard deviation of the frequency of MRB per location in the four seasons. Different alphabets represent significant differences in the frequency of MRB between seasons. 40 locations were used in the study.

We sampled an average of 15 dogs per location, considering all 40 locations for four seasons, where the dog numbers varied from 559 (monsoon) to 645 (spring) for the 40 locations taken together (S1 and S2 Figs). There was significant variation in dog numbers between seasons (Repeated measures ANOVA: F_10,309_ = 2.793, p = 0.003). However, the sex ratio did not deviate from 1:1 across the seasons (T test for each season, p < 0.05 for each season). We carried out a Kruskal-Wallis test for the frequency of MRB observed in the four seasons over the six fortnights in each season. There was significant variation between seasons, when all four seasons were considered (χ^2^ = 14.172, df = 3, p = 0.003). The significant variation was explained by the increased rate of mating related activities in the monsoon, as the significance disappeared when the three other seasons were compared (χ^2^ = 2.984, df = 2, p = 0.225), but was evident for all comparisons involving the monsoon (Fig 4).

**Fig 5:**
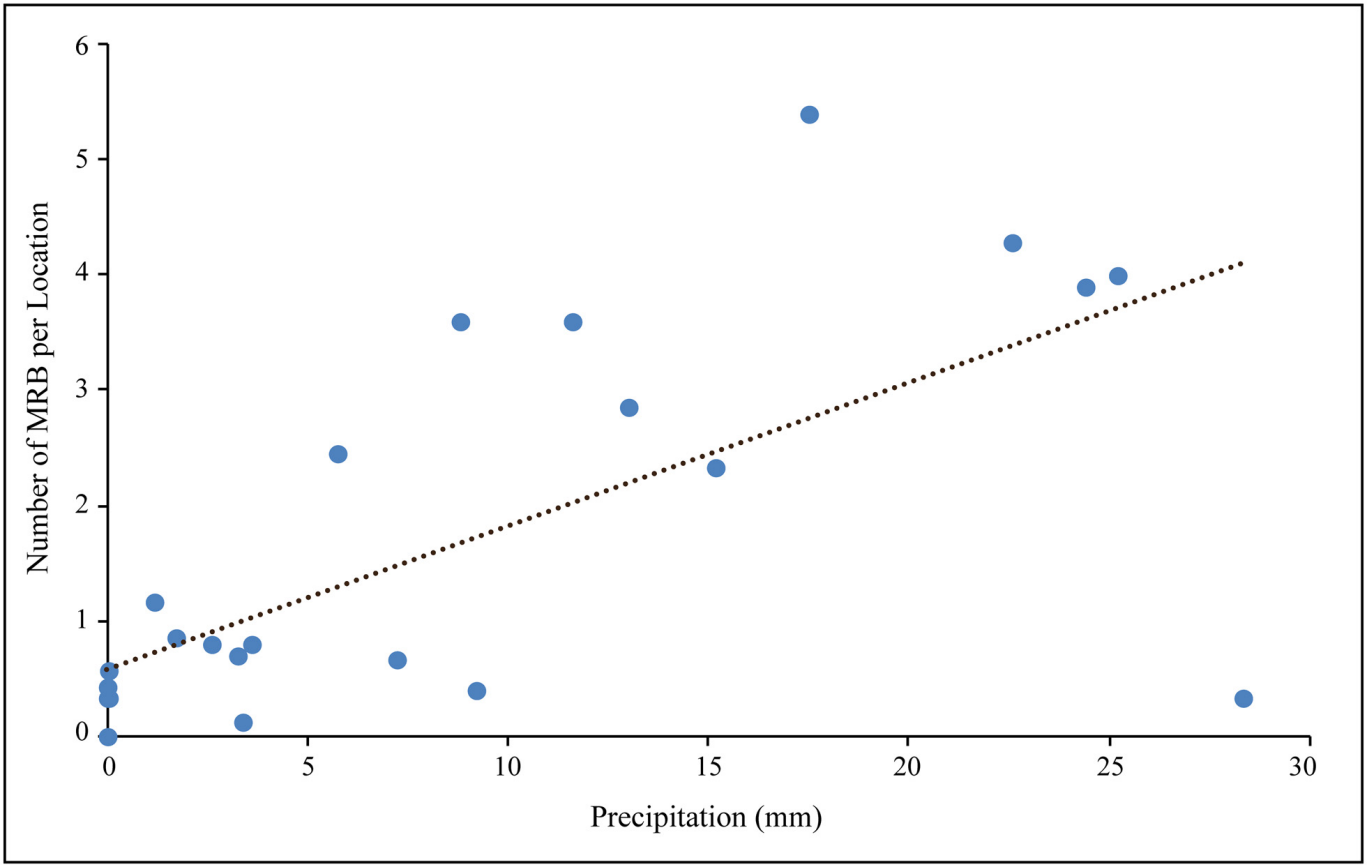
The frequency of MRB per location increased with increased precipitation. A scatterplot showing the significant interaction between the numbers of MRB observed per location at different levels of precipitation (as recorded from IMD). The dotted line shows the linear fit, and is represented by the equation y = 0.1243x + 0.581, R^2^ = 0.4704.

**Fig 6:**
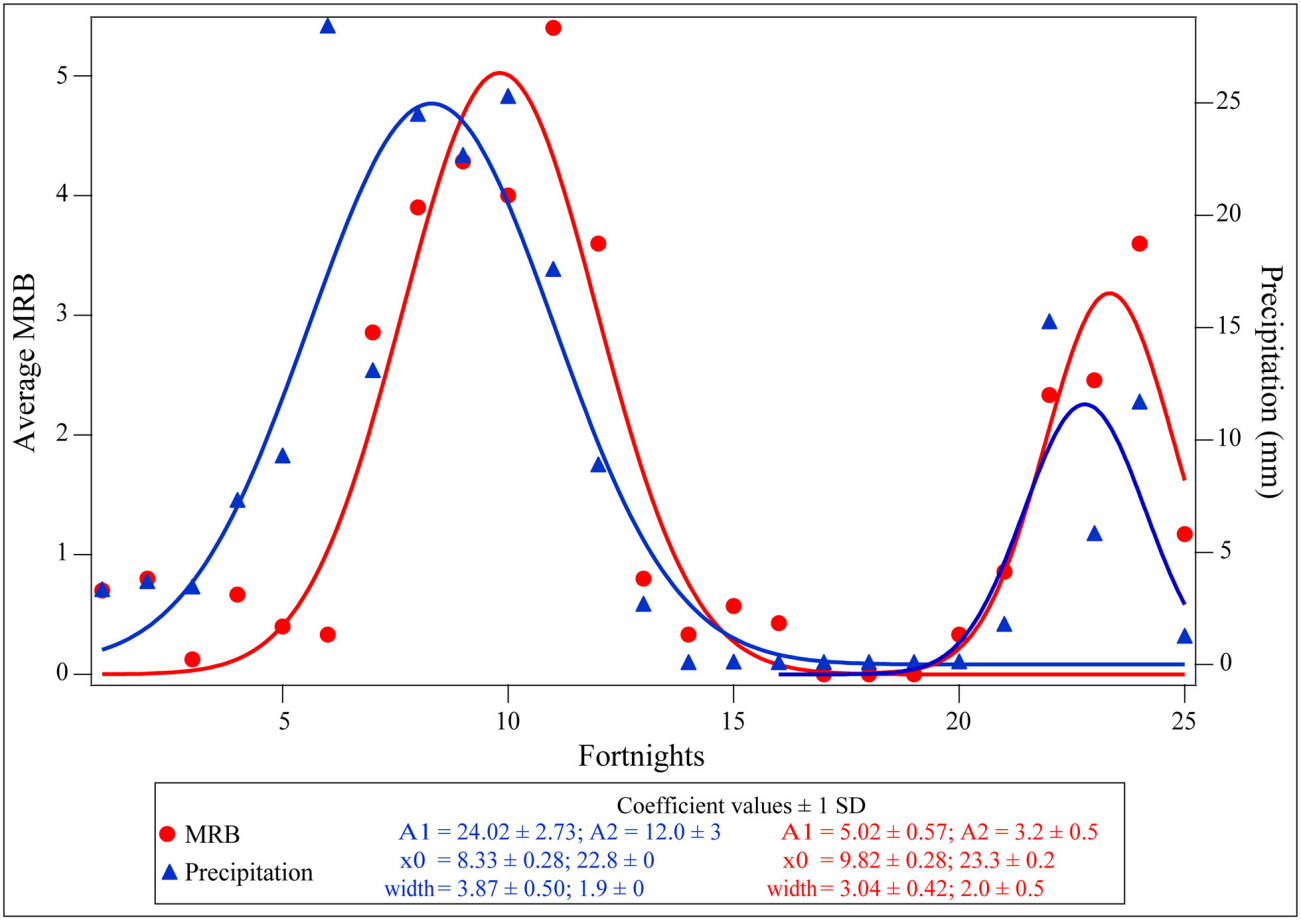
MRB and precipitation patterns show convergent double binomial trends. The plot shows the relationship between the occurrence of mating related behaviours (MRB) and precipitation levels over a year, on a fortnightly basis. The red dots represent the number of MRB averaged over the number of locations sampled in a fortnight and blue dots represent the average precipitation level (from IMD) considering only the days of the census. Both the datasets fit double normal distributions, as represented by the lines of the respective colours. A1 and A2 represent the two amplitudes of the respective curves at x0 time as read from the x-axis.

The frequency of MRB observed per location within a fortnight strongly depended on the average precipitation level in that fortnight (Linear regression: R^2^ = 0.470, F = 20.428, p < 0.0001; Fig 5). The fortnightly data for precipitation levels and frequency of observed MRB fitted double normal distributions, and there was an offset in both the sets of peaks for the two distributions (Fig 6). This confirmed unambiguously the close correlation between precipitation levels and mating activities of dogs.

## Discussion

Reproductive activity in free-ranging dogs of West Bengal is not only seasonal, but shows spectacular concordance with precipitation levels. Our results show a strong correlation between precipitation levels and the occurrence of mating related behaviours in dogs, leading to a primary mating season during the monsoon and a secondary mating season that coincides with the Norwesters or “Kal Baisakhis” in West Bengal [55], as revealed by our year-long census. While the census based study revealed the strong connection between mating activities of dogs and rainfall, the long term study of mating during the monsoon showed that there is a high degree of consistency in the mating activities over the four years of the study, at least during the primary mating season. It was interesting to note that the only deviation occurred when we compared our observation for 2013 with the remaining data, keeping the calendar month constant and ignoring the actual onset of rains. This difference vanished when we considered the data from the actual onset of rains, and was consistent with the fact that there was significant variation in MRB between the different weeks of observations within the three-month period of the monsoons.

In this study, we were interested in understanding the extent to which free-ranging dogs show seasonality of mating, and attempting to explore the plausible explanations for this behaviour. Our observations on free-ranging dogs suggest that their attempts at mating are often disrupted by people, mostly due to socio-cultural reasons, and this reduces the probability of seeing successful ties on the streets. Hence we used all activities that pertain to mating in the dogs, and not just ties, but did not use mating related aggression for this analysis. We used multivariate statistics to gain a more detailed understanding of the relationship between the mating behavioural repertoire of the dogs and rainfall. Our observations revealed that all mating related behaviours are affected by rainfall, but the fact that mounting (MT) occurs at significantly higher rates at high precipitation levels only, suggests that precipitation levels might eventually drive mating success. But why is mating in dogs influenced so intensely by precipitation levels?

The answer to this question might lie in chemistry, rather than biology. Odor is a generic term used for any compound or mixture of compounds that can be detected by animals through olfaction. Though the word odor is usually associated with unpleasant smells, technically, even pleasant smells are odors. The ability to detect odors varies greatly among species, and dogs are known for their highly developed olfactory acuity. They can detect odorant concentration levels at 1–2 parts per trillion, which makes them 10^4^–10^5^ times more efficient in sensing odors than humans [57, 58]. This fantastic sense of smell was probably one of the major factors that made dogs useful to early humans as excellent hunting companions, and also makes them indispensable in today’s world. Sniffer dogs are used for tasks as diverse as tracking criminals, locating survivors at sites of disasters, unearthing explosives and narcotics and even estimating animal populations from scat samples [59].

The efficiency of dogs at tracking and locating is known to be influenced by environmental factors like temperature and humidity. Tracking dogs are able to pick up trails better that are laid on moist, rather than dry soil, and dogs trained for hunting birds are most efficient when the ground is damp. Search-and-rescue dogs also perform better when the relative humidity is high [60]. Humans as well as birds continuously slough off dead skin containing live bacteria. The bacteria metabolize the dead cells and associated body secretions, leading to the formation of odor, which is used by dogs for tracking. High temperature and dry conditions can kill bacteria and prevent odor formation, while very cold conditions retard bacterial action, and thus decay rate. A light rain after a dry period can induce bacterial growth and hence the production of odor. Thus humidity but not very high rains increase the efficiency of dogs in tracking and searching tasks by increasing odor intensity [61]. Dogs also perform better at picking up trails when the ground is warmer than the air, because the odor rises upwards in the cooler air [60]. Olfactory function in humans have also been shown to be affected by environmental conditions—olfactory sensitivity thresholds are low in high humidity and low atmospheric pressure [62].

Ion mobility spectrometry (IMS) is a widely used technique used for detecting trace quantities of gaseous organic compounds at atmospheric pressure, and is widely used for detecting noxious elements [63–66]. One major problem associated with this technique is the presence of moisture, and currently a great deal of research focuses on dealing with this issue [67, 68]. Humidity affects the detection process in IMS by reducing both selectivity and sensitivity [69], as water interacts with ions to cause shift in peaks or the formation of additional peaks [70]. Odoriferous molecules also tend to interact with water vapour in the atmosphere, and this slows down their diffusion in the air, and humidity reduces the threshold level of olfactory sensitivity [62].

We speculate that the increased mating related activities of dogs during the monsoon is brought about by a three-pronged effect. Firstly, heavy showers serve to wash away odors present in the air so that there is less olfactory “noise” immediately after a shower. Due to the increased humidity, the sex pheromones released by the individuals diffuse slowly, and thus can be perceived in high concentration around the individuals. In contrast to the IMS instruments, the dogs perform better under reduced temperature and increased humidity conditions, which ensures that the higher concentration of sex pheromones in the air triggers higher mating related behaviours. The offset between peaks for highest precipitation MRB level probably arises because extremely heavy rains can also wash away the sex pheromones, thereby reducing their efficacy.

The absence of seasonality of reproduction in pet dogs [50] is likely to have arisen from the availability of abundant resources [1]. Most canids show strong seasonality of reproduction in the wild, the breeding season being typically determined by resource availability [27, 35, 37]. Since the free-ranging dogs are scavengers, the weather-induced seasonality of mating is likely to increase competition for resources during the breeding season, rather than ensuring resource abundance. It has been shown that male dogs get sexually excited on perceiving the urine and vaginal discharge of females in heat [71], and they also sniff and lick the anogenital regions of females in the proestrous stage [72]. Though male dogs find “estrous urine” to be an attractant, such urine loses its attractiveness when stored for an hour at room temperature. This suggests that the sex pheromone, if present, is not very stable, and is perhaps quite volatile [72]. It should be noted that sexual activity in females also increases when they are in heat, leading to increased investigation of male dogs and their urine [71]. Hence it seems likely that the increased sexual activity of dogs immediately after a shower is triggered by the increased concentration of odorants in the humid air due to a slower rate of diffusion. This would be further enhanced by the increased sensitivity to odor in humid conditions, for a short period of time while the cues from the proestrous females persist. Though such weather-induced mating behaviour can effectively increase the probability of mating success simply by increased mating attempts, it could also be effectual as a natural population control mechanism of the free-ranging dogs, due to the increase in competition for resources.

## Supporting Information

S1 Fig A map showing the position of the area where the long term behavioural observations on mating were carried out. The position of each group is marked with a yellow tag.
(JPG)

S2 Fig A map showing the 40 locations in which the year-long census was conducted pointed out using yellow tags. (JPG)

S1 Table A table giving the results of the ANOVAs conducted at the level of each mating related behaviour for variation across years at different levels of precipitation. (DOC)

## References

[1] Bronson FH. Mammalian reproductive biology, pp. 51–52 Chicago, IL: The University of Chicago Press; 1989.

[2] Bronson FH, Heideman PD. Seasonal regulation of reproduction in mammals In The physiology of reproduction (eds Knobil E., Neill J. D.), 2nd edn New York, NY: Raven Press; 1994.

[3] Prendergast B. Internalization of seasonal time. Horm Behav. 2005; 48: 503–511. 16026787 10.1016/j.yhbeh.2005.05.013

[4] Bronson FH. Seasonal variation in human reproduction: environmental factors. Quart. Rev. Biol. 1995; 70: 141–164. 7610233 10.1086/418980

[5] Brockman DK, van Schaik CP. Seasonality in primates: Studies of living and extinct human and non-human primates. Cambridge University Press; 2005.

[6] Kumar BS, Kumar V. Seasonal reproduction in subtropical brahminy myna, *Sturnus pagodarum*: role of photoperiod. Gen Comp Endocr. 1991; 83: 354–365. 1936916 10.1016/0016-6480(91)90140-2

[7] Chandola-Saklani A, Thapliyal A, Negi K, Diyundi SC, Choudhary B. Daily increments of light hours near vernal equinox synchronize circannual testicular cycle of tropical spotted munia. Chronobiol Int. 2004; 21: 553–569. 15470954 10.1081/cbi-200025991

[8] Hart RC. Cladoceran periodicity patterns in relation to selected environmental factors in two cascading warm water reservoirs over a decade. Hydrobiologia. 2004; 526: 99–117

[9] Patterson JW. Rainfall and reproduction in females of the tropical lizard *Mabuya striata striata* . Oecologia. 1991; 86: 419–423. 28312931 10.1007/BF00317611

[10] Lampo M, Medialdea V. Energy allocation patterns in *Bufo marinus* from two habitats in Venezuela. J Trop Ecol. 1996; 12: 321–331.

[11] Tinney GM, Bernard RTF, White RM. Influences of food quality and quantity on the male reproductive organs of a seasonally breeding rodent, the pouched mouse *(Saccostomus campestris*), from a seasonal but unpredictable environment. Afr Zool. 2001; 36: 23–30.

[12] Clouet M. Bill size and breeding period of pine forest crossbills. Rev Ecol-Terre Vie. 2003; 58: 419–433.

[13] Rubenstein DR, Wikelski M. Seasonal changes in food quality: a proximate cue for reproductive timing in marine iguanas. Ecology. 2003; 84: 3013–3023.

[14] Janson C, Verdolin J. Seasonality of primate births in relation to climate. Cambridge University Press; 2005.

[15] Lancaster JB, Lee RB. The annual reproductive cycle in monkeys and apes In: Primate Behavior: Field Studies of Monkeys and Apes, De Vore I. (ed.), Holt, Rinehart & Winstone, New York 1965; pp. 486–513.

[16] Janzen DH. Why mountain passes are higher in the tropics. Am Nat. 1967; 101(919): 233–249.

[17] Altmann J. Baboon mothers and infants. Harvard University Press, Cambridge; 1980.

[18] Di Bitetti MS, Janson CH. When will the stork arrive? Patterns of birth seasonality in neotropical primates. Am J Primatol. 2000; 50(2): 109–130. 10676708 10.1002/(SICI)1098-2345(200002)50:2<109::AID-AJP2>3.0.CO;2-W

[19] Pankhurst NW, Porter MJR. Cold and dark or warm or light: variations on the theme of environmental control of reproduction. Fish Physiol Biochem. 2003; 28: 385–389. doi: 10.1023/B:FISH.00000 30602.51939.50

[20] Brockman DK, van Schaik CP. Seasonality and reproductive function In Brockman D. K. & van Schaik C. P. (Eds.), Seasonality in primates: Studies of living and extinct human and nonhuman primates. Cambridge: Cambridge University Press; 2005 pp. 269–306.

[21] Tecot S. It’s all in the timing: Out of season births and infant survival in *Eulemur rubriventer* . Int J Primatol. 2000; 31(5): 715–735. 10.1007/s10764-010-9423-5

[22] Hall-Martin J, Skinner JD, Vandyk JM. Reproduction in the giraffe in relation to some environmental factors. Afr J Ecol. 1975; 13(3–4): 237–248.

[23] Smith NS, Buss IO. Reproductive Ecology of the Female African Elephant. J Wildlife Manage. 1973; 37(4): 524–534.

[24] Bodenheimer FS. Problems of physiology and ecology in desert animals Biology of Desert, Institute of Biology, London; 1954 pp. 162–167.

[25] Vandenbergh JG, Vessey S. Seasonal breeding of free-ranging rhesus monkeys and related ecological factors. J Reprod Fertility. 1968; 15: 71–79. 10.1530/jrf.0.0150071 4966619

[26] Rabb GB, Woolpy JH, Ginsburg BE. Social relationships in a group of captive wolves. Am. Zool. 1967; 7:305–311.

[27] Seager SWJ, Demorest CN. Reproduction in captive wild carnivores Zoo and Wild Animal Medicine. 2nd edn Ed Fowler M. E.. Philadelphia, W. B. Saunders 1986; pp 852–881.

[28] Mayer W. Physiological Mammalogy, Elsevier 2012; Volume 2. ISBN: 978-0-12-395674-3.

[29] Trut L, Oskina I, Kharlamova A. Animal evolution during domestication: the domesticated fox as a model. Bioessays. 2009; 31(3): 349–360. 10.1002/bies.200800070 19260016 PMC2763232

[30] Prakash I. Breeding of Mammals in Rajasthan Desert, India. J Mammal. 1960; 41(3): 386–389.

[31] Martin GB, Walkden-Brown SW. Nutritional influences on reproduction in mature male sheep and goats. J Reprod Fertil. 1995; 49: 437–449. 7623333

[32] Rubenstein DR, Wikelski M. Seasonal changes in food quality: A cue for reproductive timing in marine Iguanas. Ecology. 2003; 84:3013–3023.

[33] Arlettaz R, Jones G, Racey PA. Effect of acoustic clutter on prey detection by bats. Nature. 2001; 414: 742–745. 11742397 10.1038/414742a

[34] Holekamp KE, Szykman M, Boydston EE, Smale L. Association of seasonal reproductive patterns with changing food availability in an equatorial carnivore, the spotted hyaena *(Crocuta crocuta)* . J Reprod Fertil. 1999, 116: 87–93. 10505059 10.1530/jrf.0.1160087

[35] Hamlett GWD. The reproductive cycle of the coyote. U.S.D.A. Tech. Bull. 1938; 616:1–11.

[36] Skinner JD, Moss DG, Skinner DC. Inherent seasonality in the breeding seasons of African mammals: evidence from captive breeding. 2002; 57(1/2): 25–34.

[37] Adlerton D. Foxes, Wolves and wild Dogs of the World. New York: Facts on File; 1994 192 pp.

[38] Engle ET. No Seasonal Breeding Cycle in Dogs. J Mammal. 1946; 27(1): 79–81. 21020478

[39] Ruvinsky A, Sampson J. The genetics of the dogs. CABI Publishing; 2001. ISBN: 0-85199-520-9.

[40] Beaver BV. Canine Behavior Insights and Answers 2nd Edition Saunders, Elsevier; 2009. ISBN: 978-1-4160-5419-1.

[41] Ortega-Pacheco A, Rodríguez-Buenfil JC, Segura-Correa JC, Bolio-Gonzalez ME, Jiménez-Coello M, Linde Forsberg C. Pathological Conditions of the Reproductive Organs of Male Stray Dogs in the Tropics: Prevalence, Risk Factors, Morphological Findings and Testosterone Concentrations. Reprod domest anim. 2006; 41(5): 10.1111/j.1439-0531.2006.00688.x 16984349

[42] Beck AM. The Ecology of Stray Dogs: A Purdue University Press Study of Free-ranging Urban Animals; 1973. ISBN1557532451, 9781557532459

[43] Vanak AT, Gompper ME. Dietary niche separation between sympatric free-ranging domestic dogs and Indian foxes in central India. J Mammal. 2009; 90: 1058–1065.

[44] Vanak AT, Thaker M, Gompper ME. Experimental examination of behavioural interactions between free-ranging wild and domestic canids. Behav Ecol Sociobiol. 2009; 64:279–287.

[45] Pal SK. Mating system of free-ranging dogs (*Canis familiaris*). International Journal of Zoology; 2001.

[46] Pal SK. Population ecology of free-ranging urban dogs in West Bengal, India. Acta Theriol. 2001; 46(2): 69–78.

[47] Balakrishnan M, Sreedevi MB. Husbandry and management of the Small Indian Civet *Viverricula indica* (É. Geoffroy Saint-Hilaire, 1803) in Kerala, India. Small Carniv Conserv. 2007; 36.

[48] Solanki GS, Zothansiama. Infanticide in captive stump-tailed macaques (*Macaca arctoides*) is in accordance with the sexual selection hypothesis. Curr Sci India, 2013; 104(8).

[49] Bhadra A, Bhattacharjee D, Paul M, Singh A, Shrestha P, Bhadra A. The meat of the matter: A rule of thumb for scavenging dogs? Ethol Ecol Evol; 2015; 10.1080/03949370.2015.1076526

[50] Lord K, Feinstein M, Smith B, Coppinger R. Variation in reproductive traits of members of the genus Canis with special attention to the domestic dogs (*Canis familiaris*). Behave Process. 2013; 92:131–142. 10.1016/j.beproc.2012.10.009 23124015

[51] Sen Majumder S, Bhadra A, Ghosh A, Mitra S, Bhattacharjee D, Chatterjee J, et al To be or not to be social: foraging associations of free-ranging dogs in an urban ecosystem. Acta Ethol. 2014; 17(1): 1–8.

[52] Paul M, Sen Majumder S, Bhadra A. Selfish mothers? An empirical test of parent-offspring conflict overextended parental care. Behave Process. 2014; 103:17–22. 10.1016/j.beproc.2013.10.006 24216083

[53] Paul M, Sen Majumder S, Bhadra A. Grandmotherly care: a case study in Indian free ranging dogs. J Ethol. 2014; 32:75–82.

[54] Wang B, Linho. Rainy Season of the Asian–Pacific Summer Monsoon. J Climate. 2002; 15: 386–398.

[55] Attri SD, Tyagi A. “Climate Profile of India”. Met. Monograph Environmental Meteorology No 1/2010; 2010. pp. 1–122.

[56] Joseph PV. Monsoon monograph Vol I, Ed Tyagi et al, India Meteorological Dept; 2012 pp. 284.

[57] Tripp AC, Walker JC. The great chemical residue detection debate: Dog versus machine. *Proc*. *SPIE* 5089, Detection and Remediation Technologies for Mines and Minelike Targets VIII, 983; 2003 10.1117/12.485637

[58] Yeomans MR, Mobini S, Elliman TD, Walker HC, Stevenson RJ. Hedonic and sensory characteristics of odors conditioned by pairing with tastants in humans. J Exp Psychol Anim B. 2006; 32(3): 215–228. 10.1037/0097-7403.32.3.215 16834490

[59] Ensminger JJ. Police and Military Dogs: Criminal Detection, Forensic Evidence, and Judicial Admissibility. CRC Press. Taylor and Francis Group; 2012.

[60] Conover MR. Predator-Prey Dynamics: The Role of Olfaction. CRC Press; 2007. ISBN 1420009125, 9781420009125

[61] Gutzwiller KJ. Minimizing Dog-Induced Biases in Game Bird Research. Wildlife Soc B. 1990; 18(3): 351–356.

[62] Kuehn M, Welsch H, Zahnert T, Hummel T. Changes of pressure and humidity affect olfactory function. Eur Arch Otorhinolaryngol. 2008; 265(3): 299–302. 17901967 10.1007/s00405-007-0446-2

[63] Hill HH Jr, Siems WF, St. Louis RH. Ion mobility spectrometry. Anal. Chem. 1990; 62(23): 1201A–1209A. 10.1021/ac00222a001 2288410

[64] St. Louis RH, Hill HH Jr, Eiceman GA. Ion Mobility Spectrometry in Analytical Chemistry. pp. 321–355. Reviews in Anal Chem. 1990; 21(5). 10.1080/10408349008050848 2288410

[65] Xie Z, St Sielemann S, Schmidt H, Baumbach JI. A novel Method for the Detection of MTBE: Ion Mobility Spectrometry coupled to Multi Capillary Column. IJIMS 4. 2000(1); 77–83: 78.

[66] Perl T, Carstens E, Quintel M, Vautz W, Nolte J, Jünger M. Determination of serum propofol concentrations by breath analysis using ion mobility spectrometry. Brit J Anaesth; 2009 10.1093/bja/aep312 19887534

[67] Hill HH, Simpson G. Capabilities and Limitations of Ion Mobility Spectrometry for Field Screening Applications. Field anal Chem Tech; 1997.

[68] Vautza W, Sielemannb S, Baumbacha JI. Determination of terpenes in humid ambient air using ultraviolet ion mobility spectrometry. Analytica Chimica Acta. 2004; 513(2): 393–399. 10.1016/j.aca.2004.03.016

[69] Mäkinen M, Nousiainen M, Sillanpää M. Ion spectrometric detection technologies for ultra-traces of explosives: A review. Mass Spectrom Rev. 2011; 30(5): 940–973. 10.1002/mas.20308 21294149

[70] Eiceman GA, Karpas Z, Hill HH Jr. Ion Mobility Spectrometry, Third Edition CRC Press; 2014.

[71] Dunbar IF. Olfactory preferences in dogs: the response of male and female beagles to conspecific odors. Behavioral Biology. 1977; 20(4): 471–481. 561600 10.1016/s0091-6773(77)91079-3

[72] Spotte S. Societies of wolves and free-ranging dogs. Cambridge University Press; 2012.

