# Supplementary figures and images for: When Love Is in the Air: Understanding Why Dogs Tend to Mate when It Rains
Source: PLoS One. 2015 Dec 2;10(12):e0143501. doi: 10.1371/journal.pone.0143501 (PMC4668084; doi:10.1371/journal.pone.0143501)

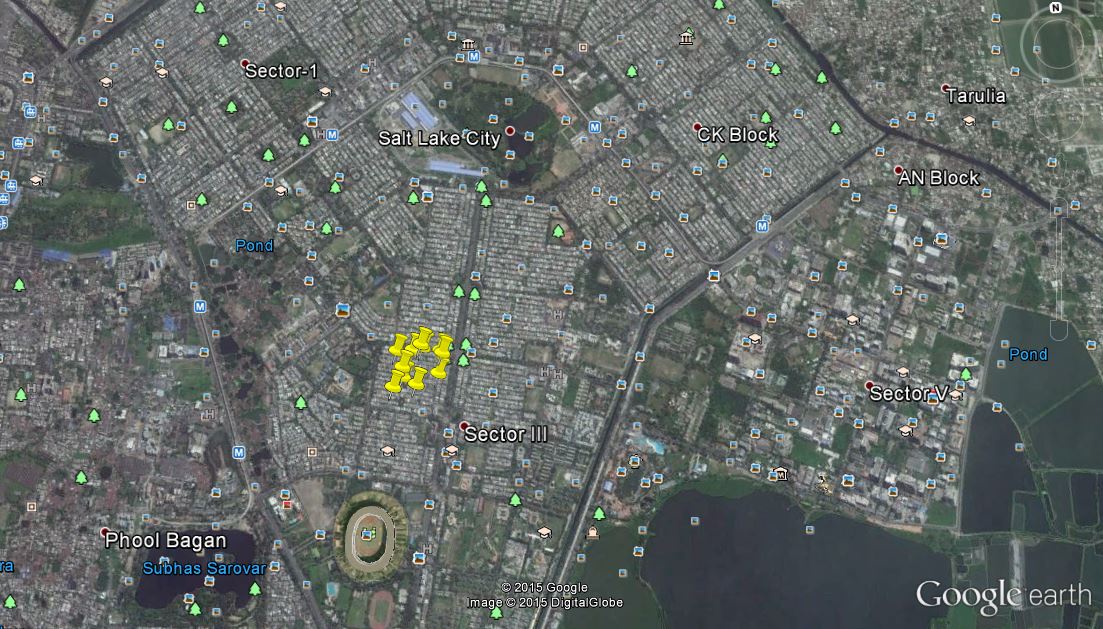

Supplement: S1 Fig — The position of each group is marked with a yellow tag. (JPG) [file pone.0143501.s001.JPG]

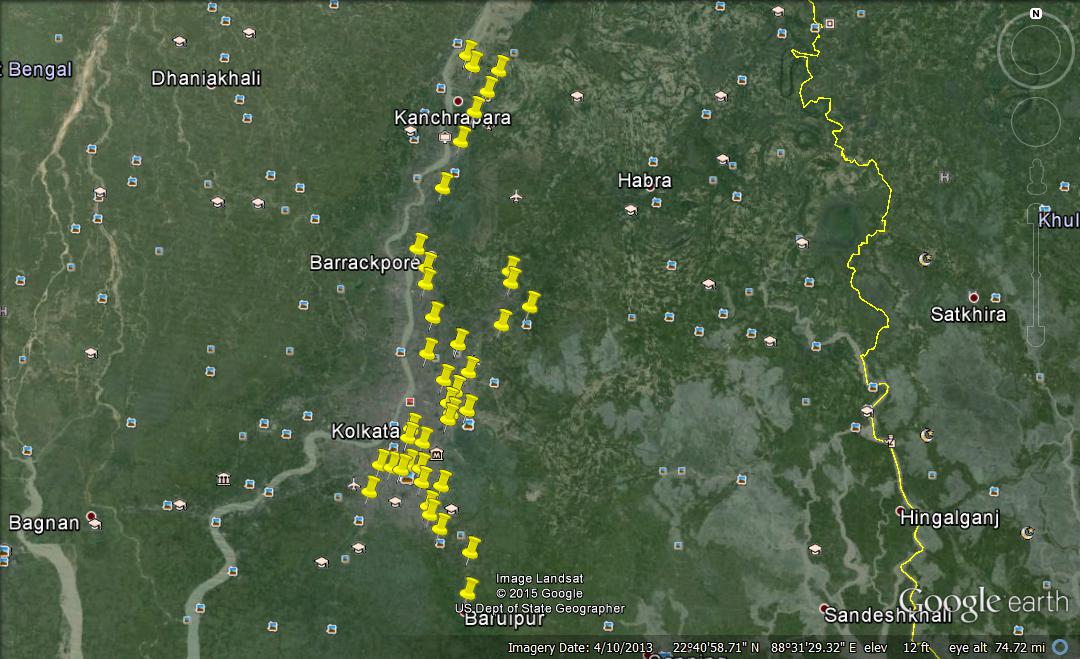

Supplement: S2 Fig — (JPG) [file pone.0143501.s002.jpg]
