# Supplementary material for: When Love Is in the Air: Understanding Why Dogs Tend to Mate when It Rains
Source: PLoS One. 2015 Dec 2;10(12):e0143501. doi: 10.1371/journal.pone.0143501 (PMC4668084; doi:10.1371/journal.pone.0143501)
Supplement: S1 Table — (DOC) [file pone.0143501.s003.doc]

**When love is in the air: Understanding why dogs tend to mate when it rains**

**Supplementary Information – S1 Table**

**Try to Clasp (TC)**

| **Overall test of model for Y=TC** | | |  |  |  |  |
| --- | --- | --- | --- | --- | --- | --- |
| Source | Type III SS | Df | Mean Sq. | F | Prob. |  |
| Model | 2243.667 | 11 | 203.970 | 24.118 | 0.000 |  |
| Error | 1116.333 | 132 | 8.457 |  |  |  |
| Total | 3360.000 | 143 |  |  |  |  |
|  |  |  |  |  |  |  |
| **Tests of effects for Y=TC** | | |  |  |  |  |
| Source | Type III SS | Df | Mean Sq. | F | Prob. |  |
| Rainfall | 2035.542 | 2 | 1017.771 | 120.346 | 0.000 |  |
| Year | 65.000 | 3 | 21.667 | 2.562 | 0.058 |  |
| Rainfall*Year | 143.125 | 6 | 23.854 | 2.821 | 0.013 |  |
|  |  |  |  |  |  |  |
|  |  |  |  |  |  |  |
| **Post Hoc tests for Factor = Rainfall** | | | |  |  |  |
| Test | Group 1 | Group 2 | Mean Diff. | SE | q | Prob. |
| Tukey | High | Medium | 6.771 | 0.420 | 16.131 | 0.000 |
|  |  | Nill | 8.792 | 0.420 | 20.945 | 0.000 |
|  | Medium | Nill | 2.021 | 0.420 | 4.814 | 0.002 |
| Scheffe | High | Medium | 6.771 | 0.594 | 11.406 | 0.000 |
|  |  | Nill | 8.792 | 0.594 | 14.810 | 0.000 |
|  | Medium | Nill | 2.021 | 0.594 | 3.404 | 0.004 |
|  |  |  |  |  |  |  |
| **Post Hoc tests for Factor = Year** | | | |  |  |  |
| Test | Group 1 | Group 2 | Mean Diff. | SE | q | Prob. |
| Tukey | 2010 | 2011 | -0.389 | 0.485 | 0.802 | 0.942 |
|  |  | 2012 | -0.889 | 0.485 | 1.834 | 0.565 |
|  |  | 2013 | 0.944 | 0.485 | 1.949 | 0.513 |
|  | 2011 | 2012 | -0.500 | 0.485 | 1.032 | 0.885 |
|  |  | 2013 | 1.333 | 0.485 | 2.751 | 0.209 |
|  | 2012 | 2013 | 1.833 | 0.485 | 3.783 | 0.038 |
| Scheffe | 2010 | 2011 | -0.389 | 0.685 | 0.567 | 0.956 |
|  |  | 2012 | -0.889 | 0.685 | 1.297 | 0.642 |
|  |  | 2013 | 0.944 | 0.685 | 1.378 | 0.595 |
|  | 2011 | 2012 | -0.500 | 0.685 | 0.729 | 0.912 |
|  |  | 2013 | 1.333 | 0.685 | 1.945 | 0.290 |
|  | 2012 | 2013 | 1.833 | 0.685 | 2.675 | 0.072 |

| **Genital sniffing (GS)**  **Overall test of model for Y=GS** | | |  |  |  |  |
| --- | --- | --- | --- | --- | --- | --- |
| Source | Type III SS | Df | Mean Sq. | F | Prob. |  |
| Model | 10046.0763888 | 11 | 913.280 | 37.138 | 0.000 |  |
| Error | 3246.083 | 132 | 24.592 |  |  |  |
| Total | 13292.15972222 | 143 |  |  |  |  |
|  |  |  |  |  |  |  |
| **Tests of effects for Y=GS** | | |  |  |  |  |
| Source | Type III SS | Df | Mean Sq. | F | Prob. |  |
| Rainfall | 9459.014 | 2 | 4729.507 | 192.323 | 0.000 |  |
| Year | 336.799 | 3 | 112.266 | 4.565 | 0.004 |  |
| Rainfall*Year | 250.264 | 6 | 41.711 | 1.696 | 0.127 |  |
|  |  |  |  |  |  |  |
|  |  |  |  |  |  |  |
| **Post Hoc tests for Factor = Rainfall** | | | |  |  |  |
| Test | Group 1 | Group 2 | Mean Diff. | SE | q | Prob. |
| Tukey | High | Medium | 14.333 | 0.716 | 20.025 | 0.000 |
|  |  | Nill | 19.063 | 0.716 | 26.632 | 0.000 |
|  | Medium | Nill | 4.729 | 0.716 | 6.607 | 0.000 |
| Scheffe | High | Medium | 14.333 | 1.012 | 14.160 | 0.000 |
|  |  | Nill | 19.063 | 1.012 | 18.832 | 0.000 |
|  | Medium | Nill | 4.729 | 1.012 | 4.672 | 0.000 |
|  |  |  |  |  |  |  |
| **Post Hoc tests for Factor = Year** | | | |  |  |  |
| Test | Group 1 | Group 2 | Mean Diff. | SE | q | Prob. |
| Tukey | 2010 | 2011 | -0.583 | 0.826 | 0.706 | 0.959 |
|  |  | 2012 | -1.972 | 0.826 | 2.386 | 0.330 |
|  |  | 2013 | -3.972 | 0.826 | 4.806 | 0.004 |
|  | 2011 | 2012 | -1.389 | 0.826 | 1.680 | 0.634 |
|  |  | 2013 | -3.389 | 0.826 | 4.100 | 0.020 |
|  | 2012 | 2013 | -2.000 | 0.826 | 2.420 | 0.318 |
| Scheffe | 2010 | 2011 | -0.583 | 1.169 | 0.499 | 0.969 |
|  |  | 2012 | -1.972 | 1.169 | 1.687 | 0.419 |
|  |  | 2013 | -3.972 | 1.169 | 3.398 | 0.011 |
|  | 2011 | 2012 | -1.389 | 1.169 | 1.188 | 0.703 |
|  |  | 2013 | -3.389 | 1.169 | 2.899 | 0.042 |
|  | 2012 | 2013 | -2.000 | 1.169 | 1.711 | 0.406 |

| **Marking with urine (MK)**  **Overall test of model for Y=MK** | | | |  |  |  |
| --- | --- | --- | --- | --- | --- | --- |
| Source | Type III SS | Df | Mean Sq. | F | Prob. |  |
| Model | 959.639 | 11 | 87.240 | 24.554 | 0.000 |  |
| Error | 469.000 | 132 | 3.553 |  |  |  |
| Total | 1428.639 | 143 |  |  |  |  |
|  |  |  |  |  |  |  |
| **Tests of effects for Y=MK** | | |  |  |  |  |
| Source | Type III SS | Df | Mean Sq. | F | Prob. |  |
| Rainfall | 882.681 | 2 | 441.340 | 124.215 | 0.000 |  |
| Year | 49.139 | 3 | 16.380 | 4.610 | 0.004 |  |
| Rainfall*Year | 27.819 | 6 | 4.637 | 1.305 | 0.259 |  |
|  |  |  |  |  |  |  |
|  |  |  |  |  |  |  |
| **Post Hoc tests for Factor = Rainfall** | | | |  |  |  |
| Test | Group 1 | Group 2 | Mean Diff. | SE | q | Prob. |
| Tukey | High | Medium | 4.500 | 0.272 | 16.540 | 0.000 |
|  |  | Nill | 5.771 | 0.272 | 21.211 | 0.000 |
|  | Medium | Nill | 1.271 | 0.272 | 4.671 | 0.003 |
| Scheffe | High | Medium | 4.500 | 0.385 | 11.695 | 0.000 |
|  |  | Nill | 5.771 | 0.385 | 14.998 | 0.000 |
|  | Medium | Nill | 1.271 | 0.385 | 3.303 | 0.005 |
|  |  |  |  |  |  |  |
| **Post Hoc tests for Factor = Year** | | | |  |  |  |
| Test | Group 1 | Group 2 | Mean Diff. | SE | q | Prob. |
| Tukey | 2010 | 2011 | 1.417 | 0.314 | 4.509 | 0.008 |
|  |  | 2012 | 0.444 | 0.314 | 1.415 | 0.749 |
|  |  | 2013 | -0.028 | 0.314 | 0.088 | 1.000 |
|  | 2011 | 2012 | -0.972 | 0.314 | 3.095 | 0.126 |
|  |  | 2013 | -1.444 | 0.314 | 4.598 | 0.006 |
|  | 2012 | 2013 | -0.472 | 0.314 | 1.503 | 0.712 |
| Scheffe | 2010 | 2011 | 1.417 | 0.444 | 3.189 | 0.020 |
|  |  | 2012 | 0.444 | 0.444 | 1.000 | 0.801 |
|  |  | 2013 | -0.028 | 0.444 | 0.063 | 1.000 |
|  | 2011 | 2012 | -0.972 | 0.444 | 2.188 | 0.193 |
|  |  | 2013 | -1.444 | 0.444 | 3.251 | 0.017 |
|  | 2012 | 2013 | -0.472 | 0.444 | 1.063 | 0.770 |

| **Running together (RT)**  **Overall test of model for Y=RT** | | |  |  |  |  |
| --- | --- | --- | --- | --- | --- | --- |
| Source | Type III SS | Df | Mean Sq. | F | Prob. |  |
| Model | 5739.688 | 11 | 521.790 | 36.018 | 0.000 |  |
| Error | 1912.250 | 132 | 14.487 |  |  |  |
| Total | 7651.938 | 143 |  |  |  |  |
|  |  |  |  |  |  |  |
| **Tests of effects for Y=RT** | | |  |  |  |  |
| Source | Type III SS | Df | Mean Sq. | F | Prob. |  |
| Rainfall | 5248.625 | 2 | 2624.313 | 181.153 | 0.000 |  |
| Year | 260.743 | 3 | 86.914 | 6.000 | 0.001 |  |
| Rainfall*Year | 230.319 | 6 | 38.387 | 2.650 | 0.018 |  |
|  |  |  |  |  |  |  |
|  |  |  |  |  |  |  |
| **Post Hoc tests for Factor = Rainfall** | | | |  |  |  |
| Test | Group 1 | Group 2 | Mean Diff. | SE | q | Prob. |
| Tukey | High | Medium | 11.250 | 0.549 | 20.478 | 0.000 |
|  |  | Nill | 13.938 | 0.549 | 25.370 | 0.000 |
|  | Medium | Nill | 2.688 | 0.549 | 4.892 | 0.002 |
| Scheffe | High | Medium | 11.250 | 0.777 | 14.480 | 0.000 |
|  |  | Nill | 13.938 | 0.777 | 17.939 | 0.000 |
|  | Medium | Nill | 2.688 | 0.777 | 3.459 | 0.003 |
|  |  |  |  |  |  |  |
| **Post Hoc tests for Factor = Year** | | | |  |  |  |
| Test | Group 1 | Group 2 | Mean Diff. | SE | q | Prob. |
| Tukey | 2010 | 2011 | 3.306 | 0.634 | 5.211 | 0.001 |
|  |  | 2012 | 1.444 | 0.634 | 2.277 | 0.373 |
|  |  | 2013 | 3.111 | 0.634 | 4.904 | 0.003 |
|  | 2011 | 2012 | -1.861 | 0.634 | 2.934 | 0.161 |
|  |  | 2013 | -0.194 | 0.634 | 0.307 | 0.996 |
|  | 2012 | 2013 | 1.667 | 0.634 | 2.627 | 0.246 |
| Scheffe | 2010 | 2011 | 3.306 | 0.897 | 3.685 | 0.005 |
|  |  | 2012 | 1.444 | 0.897 | 1.610 | 0.462 |
|  |  | 2013 | 3.111 | 0.897 | 3.468 | 0.009 |
|  | 2011 | 2012 | -1.861 | 0.897 | 2.075 | 0.236 |
|  |  | 2013 | -0.194 | 0.897 | 0.217 | 0.997 |
|  | 2012 | 2013 | 1.667 | 0.897 | 1.858 | 0.331 |

| **Mount (MT)**  **Overall test of model for Y=MT** | | | |  |  |  |
| --- | --- | --- | --- | --- | --- | --- |
| Source | Type III SS | Df | Mean Sq. | F | Prob. |  |
| Model | 388.854 | 11 | 35.350 | 10.603 | 0.000 |  |
| Error | 440.083 | 132 | 3.334 |  |  |  |
| Total | 828.938 | 143 |  |  |  |  |
|  |  |  |  |  |  |  |
| **Tests of effects for Y=MT** | | |  |  |  |  |
| Source | Type III SS | Df | Mean Sq. | F | Prob. |  |
| Rainfall | 366.000 | 2 | 183.000 | 54.890 | 0.000 |  |
| Year | 7.299 | 3 | 2.433 | 0.730 | 0.536 |  |
| Rainfall*Year | 15.556 | 6 | 2.593 | 0.778 | 0.589 |  |
|  |  |  |  |  |  |  |
|  |  |  |  |  |  |  |
| **Post Hoc tests for Factor = Rainfall** | | | |  |  |  |
| Test | Group 1 | Group 2 | Mean Diff. | SE | q | Prob. |
| Tukey | High | Medium | 3.250 | 0.264 | 12.332 | 0.000 |
|  |  | Nill | 3.500 | 0.264 | 13.280 | 0.000 |
|  | Medium | Nill | 0.250 | 0.264 | 0.949 | 0.781 |
| Scheffe | High | Medium | 3.250 | 0.373 | 8.720 | 0.000 |
|  |  | Nill | 3.500 | 0.373 | 9.391 | 0.000 |
|  | Medium | Nill | 0.250 | 0.373 | 0.671 | 0.799 |
|  |  |  |  |  |  |  |
| **Post Hoc tests for Factor = Year** | | | |  |  |  |
| Test | Group 1 | Group 2 | Mean Diff. | SE | q | Prob. |
| Tukey | 2010 | 2011 | -0.472 | 0.304 | 1.552 | 0.691 |
|  |  | 2012 | -0.583 | 0.304 | 1.917 | 0.528 |
|  |  | 2013 | -0.472 | 0.304 | 1.552 | 0.691 |
|  | 2011 | 2012 | -0.111 | 0.304 | 0.365 | 0.994 |
|  |  | 2013 | 0.000 | 0.304 | 0.000 | 1.000 |
|  | 2012 | 2013 | 0.111 | 0.304 | 0.365 | 0.994 |
| Scheffe | 2010 | 2011 | -0.472 | 0.430 | 1.097 | 0.752 |
|  |  | 2012 | -0.583 | 0.430 | 1.355 | 0.608 |
|  |  | 2013 | -0.472 | 0.430 | 1.097 | 0.752 |
|  | 2011 | 2012 | -0.111 | 0.430 | 0.258 | 0.995 |
|  |  | 2013 | 0.000 | 0.430 | 0.000 | 1.000 |
|  | 2012 | 2013 | 0.111 | 0.430 | 0.258 | 0.995 |
